# Effect of Laparoscopic Sleeve Gastrectomy on the Pharmacokinetics of Oral Omeprazole Using a Population Approach

**DOI:** 10.3390/pharmaceutics14101986

**Published:** 2022-09-20

**Authors:** Kaifeng Chen, Ping Luo, Shaihong Zhu, Yaqi Lin, Nan Yang, Shuqi Huang, Qin Ding, Liyong Zhu, Qi Pei

**Affiliations:** 1 Department of Pharmacy, The Third Xiangya Hospital, Central South University, Changsha 410013, China; 2 Department of General Surgery, The Third Xiangya Hospital, Central South University, Changsha 410013, China

**Keywords:** laparoscopic sleeve gastrectomy, omeprazole, population pharmacokinetic, modeling and simulation, obesity

## Abstract

Omeprazole is commonly prescribed to obese patients and patients after laparoscopic sleeve gastrectomy (LSG). The pharmacokinetics of oral omeprazole after LSG are still unknown. Therefore, the aim of this study was to investigate the pharmacokinetics of oral omeprazole in obese patients before and after LSG. A total of 331 blood samples were collected from 62 obese patients preoperatively (visit 1) followed by 41 patients 7 days post-LSG (visit 2) and 20 patients 1 month post-LSG (visit 3). Population pharmacokinetic analysis was performed using NONMEM to characterize the effect of LSG on omeprazole absorption and disposition. A one-compartment model with 12 transit absorption compartments and linear elimination successfully described the data. Compared with pre-surgery, the oral omeprazole time to maximum plasma concentration (T_max_) was reduced and maximum plasma concentration (C_max_) was higher, but the apparent clearance (CL/F) and area under the plasma concentration–time curve (AUC) were unchanged 7 days and 1 month after surgery. In addition, the CYP2C19 genotype and liver function exhibited a significant influence on omeprazole CL/F. LSG increased the rate of omeprazole absorption but did not affect omeprazole exposure. A dose of 20 mg omeprazole once daily may be adequate for relieving gastrointestinal tract discomfort at short-term follow-up post-LSG.

## 1. Introduction

Obesity is defined as a body mass index (BMI) ≥ 30 kg/m^2^, and it has become a global public health concern [1]. Obesity is considered to be a strong risk factor for various diseases, such as type 2 diabetes, hyperlipidemia, hypertension, nonalcoholic fatty liver disease, and sleep apnea syndrome [2], thereby leading to a decreased life quality and average life expectancy [3]. For most patients with severe obesity, the effects of nonsurgical treatments, such as reducing food intake, increasing physical activity, and drug therapy, are still limited and reversible [4]. To achieve sustained weight loss and improve obesity-related comorbidities, bariatric surgery is undoubtedly the most effective treatment option [5,6].

Currently, laparoscopic sleeve gastrectomy (LSG) is the most popular bariatric surgery worldwide, followed by Roux-en-Y gastric bypass (RYGB) [7]. LSG is well accepted as a restrictive procedure that mainly reduces the gastric volume, leaving only a smaller tube-shaped gastric pouch [8]. RYGB involves creating a small gastric pouch and bypassing the duodenum and proximal jejunum, which combines both restrictive and malabsorptive features [8]. Compared with RYGB, LSG is less invasive and has fewer complications [9]. However, due to the removal of most of the stomach fundus and body, intragastric pressure increases, and gastric compliance decreases post-LSG [10]. As a consequence, the majority of patients suffer from gastrointestinal symptoms such as pantothenic acid, nausea, sore throat, chest pain, and epigastric pain [10]. Since proton pump inhibitors (PPIs) can reduce gastric acid secretion and protect the gastric wall barrier, obese patients undergoing bariatric surgery are instructed to take PPIs for several months to relieve these symptoms and prevent gastric ulcers [11,12,13].

Omeprazole, a first-generation PPI, is widely used for the prevention and treatment of acid-related disorders such as gastric and duodenal ulcers, gastroesophageal reflux disease, and Zollinger–Ellison syndrome [14]. Given its instability in an acidic environment, oral forms of omeprazole are usually formulated as enteric-coated preparations to prevent their early degradation in the stomach [15], e.g., as enteric-coated capsules. A previous study reported that the majority of RYGB patients receiving 40 mg of omeprazole daily to prevent gastric ulcers continued to have peptic injuries 2 months after surgery [11], suggesting that the dose was not sufficient to achieve a serum level that could effectively block the production of hydrochloric acid. Furthermore, it has been well established that LSG can increase gastric pH, accelerate gastric emptying, and reduce small intestinal transit time, etc. [16,17,18]; therefore, drug absorption and disposition might be altered after LSG surgery. However, currently, there is a research gap regarding the pharmacokinetic (PK) properties of omeprazole after LSG and, consequently, regarding its effectiveness in blocking acid secretion; hence, it is crucial to investigate the influence of LSG on omeprazole PK to recommend an appropriate omeprazole dose for patients undergoing LSG.

In this study, we prospectively collected data from 62 obese patients before surgery (visit 1), 41 patients 7 days post-LSG (visit 2), and 20 patients 1 month after LSG (visit 3). For this, a population PK model was established to evaluate the effect of LSG on the PK profile of omeprazole.

## 2. Materials and Methods

### 2.1. Study Population

We designed a prospective clinical trial to recruit obese patients who were potential candidates for LSG (BMI ≥ 30 kg/m^2^). Participants with normal liver function or mild liver dysfunction were included. Mild liver dysfunction was defined as a total bilirubin (TBIL) level > 1–1.5 fold the upper limit of normal (ULN) or alanine aminotransferase (ALT) level > ULN or aspartate aminotransferase (AST) level > ULN [19], and the ULN was defined as 20.5 μmol/L, 40 U/L and 35 U/L for TBIL, ALT, and AST according to our laboratory standards. In addition, participants were excluded if they were known to have used CYP2C19 or CYP3A4 inhibitors and inducers over a period of three visits or were allergic to omeprazole. Before inclusion, all patients signed written informed consent. This study was performed at the Third Xiangya Hospital of Central South University after obtaining approval by the Ethics Committee and registered at the China Clinical Trial Registration Center with the identifier ChiCTR2100046578. All subjects were genotyped for CYP2C19 *2 (rs4244285), *3 (rs4986893), and *17 (rs12248560) using PCR-Fluorescence Probing [20] and were classified based on the updated Clinical Pharmacogenetics Implementation Consortium (CPIC) guidelines [21].

### 2.2. Study Design and Analytical Assay

Obese patients received a single 20 mg oral dose of omeprazole enteric-coated capsules under fasting conditions 2–3 days before surgery. Subsequently, 20 mg omeprazole was orally administered once daily to relieve gastrointestinal symptoms from the 7th day after LSG to 1 month after LSG. Consequently, this PK study was performed three times: after a single dose before surgery (visit 1), after a single dose 7 days post-LSG (visit 2), and after a repeated dose 1 month post-LSG (visit 3). Blood samples were obtained at approximately 1, 2, 4, 6, 8, and 9 h after omeprazole intake. The majority of patients had three blood samples collected before surgery, three collected 7 days after LSG and two collected 1 month post-LSG. Blood samples were centrifuged for 10 min at 4 °C at 2500× *g*, after which plasma was stored at −80 °C until analysis. Plasma samples were analyzed using a validated liquid chromatogram tandem mass spectrometry method [20]. The lower limit of quantification of this assay was 1 ng/mL, and the upper limit of quantification was 2000 ng/mL. The quality control samples demonstrated that the intra-day and inter-days coefficients of variation were less than 10%.

### 2.3. Structural PK Model

A nonlinear mixed effects modeling program NONMEM (version 7.5; Icon Development Solutions, Ellicott City, MD, USA) was used to perform the population PK analysis, and R (version 4.2, http://www.r-project.org/, accessed on 6 January 2022) was used to visualize the data. The first-order conditional estimation method with inter- and intra-individual interactions (FOCE-I) was used throughout the model-building procedure. Different structural and statistical models were distinguished by means of comparing the objective function value (OFV). A *p*-value below 0.05, representing a reduction of 3.84 in the OFV, was considered statistically significant. Furthermore, goodness-of-fit (GOF) plots and visual predictive check (VPC) were used for diagnostic purposes [22]. Moreover, the accuracy and precision of the parameter estimates were used to assess the model. Sampling importance resampling (SIR) approach using 1000 replicates was used to obtain the 95% confidence intervals and to evaluate the robustness of the population PK model [23].

Initially, we tested one-compartment and two-compartment models. The delay in release of omeprazole enteric-coated capsules was captured by testing several transit compartment models. The time course of omeprazole concentrations was eventually modeled using a one-compartment model with 12 transit absorption compartments, which was consistent with our previous work [20]. The model was parameterized in terms of mean transit time (MTT) which represented the average time from oral administration of the drug to its appearance at the sampling point [24], apparent clearance (CL/F), and apparent volume of distribution (Vd/F). The inter-individual variability and inter-occasion variability (IOV) for the PK model parameters were modeled using an exponential model. A proportional residual error model was used to account for intra-individual variability. We have attached the NONMEM code as supplemental material.

### 2.4. Selection of Covariates

For evaluating potential relationships, covariates were plotted independently against the individual estimates of PK parameters. The following covariates were explored: visits, CYP2C19 genotype, liver function (normal liver function and mild liver dysfunction), sex, total body weight (TBW), age, lean body weight (LBW, based on the Janmahasatian formula [25]), ideal body weight (IBW, based on the Devine formula [26]), and adjusted body weight (ABW, based on a criterion defined by Schwartz [27]).

Continuous covariates, such as age, and TBW, were evaluated using a power function model, as presented in Equation (1).

(1) $$\;P_{i}=\theta_{p}*{\left(\frac{Cov}{Cov_{median}}\right)}^{\theta_{cov}}*e^{\eta_{j}}$$

where *P_i_* and *θ_p_* represent the individual and typical population estimates, respectively; *Cov_median_* is the median value for the covariate except that TBW was normalized to 70 kg; *θ_cov_* is the estimated influential factor for the covariate; *η_j_* is the random effect that describes the difference of the *j*th subject from the typical population value.

Categorical variables, such as visits and the CYP2C19 genotype, were assessed as follows (take visits as an example):

(2) $$\;\;\;\;\;\;\;\;\;\;\;\;\;\;\;\;\;P_{i}=\theta_{p}*e^{\eta_{j}}$$

(3) $$\;\;\;\;\;\;\;\;\;\;\;\;\;\;\;\;IF\left(Visit.EQ.2\right)\;\;P_{i}=\theta_{1}*\;P_{i}$$

(4) $$\;\;\;\;\;\;\;\;\;\;\;\;\;\;\;\;IF\left(Visit.EQ.3\right)\;\;P_{i}=\theta_{2}*P_{i}$$

where $\theta_{p}$ is the typical value for obese patients before surgery, namely, at visit 1; θ_1_ represents the numerical differences between visit 1 and visit 2 (7 days post-LSG); $\theta_{2}\;$ represents the numerical differences between visit 1 and visit 3 (1 month post-LSG).

During forward inclusion, covariates that decreased the OFV by >3.84 (1 df, *p* < 0.05) were retained for the further multivariable analysis. Afterwards, these covariates could be kept only if removal of the covariates increased the OFV by >10.83 (1 df, *p* < 0.001). In addition, a reduction in inter-subject variability was also evaluated for a given parameter. The selection of the covariate model was further assessed as discussed above (see structural PK model section).

### 2.5. Monte Carlo Simulations

The final population PK model was used to simulate the omeprazole concentration profiles at a single dose of 20 mg omeprazole before and 7 days after surgery and at a repeated dose 1 month after surgery. The Friedman nonparametric paired test was used to compare the values of T_max_ (time to maximum plasma concentration) and C_max_ (maximum plasma concentration) obtained during the three visits. These secondary parameters on two occasions (visit 2 vs. visit 1 and visit 3 vs. visit 1) were then compared using the Wilcoxon nonparametric paired test.

## 3. Results

### 3.1. Subjects and Data

In total, 62 patients with obesity were included pre-surgery, with a median BMI of 40.3 kg/m^2^ (range 30–72.8 kg/m^2^). Of the 62 patients tested at visit 1, 41 completed the PK study 7 days post-LSG (visit 2), and 20 completed the PK study 1 month post-LSG (visit 3). Table 1 shows the demographic and clinical characteristics of the included patients during the three visits. DNA samples from 62 volunteers were genotyped, and we identified 26 normal metabolizers (NMs), 27 intermediate metabolizers (IMs), and 9 poor metabolizers (PMs) according to the CPIC guidelines. A total of 331 concentration–time points were obtained for the population PK analysis: 190 concentrations at visit 1; 101 concentrations at visit 2; 40 concentrations at visit 3.

### 3.2. Population PK Modeling

Based on the data, a one-compartment model with 12 transit absorption compartments and linear elimination was identified in which visits proved a significant covariate for MTT, and the CYP2C19 genotype and liver function exhibited a remarkable effect on CL/F. In addition, the data did not support estimates of inter-individual variability in Vd/F and setting the variance of this random effect to zero did not influence the OFV.

Implementation of the visits on MTT led to a 131.5 point (*p* < 0.001) reduction in OFV, and a shorter MTT was observed 7 days and 1 month post-LSG compared with preoperatively. No significant trend was found for visits and CL/F (*p* = 0.075), although omeprazole plasma concentrations reached a steady-state 1 month after surgery compared with a single omeprazole dose before surgery and 7 days post-LSG. However, IOV had a significant effect on CL/F, decreasing residual variability from 45.6% to 41.8%. Moreover, CYP2C19 IMs and PMs showed a lower CL/F relative to NMs (ΔOFV −21; *p* < 0.001). Individuals with mild liver dysfunction exhibited a lower CL/F than normal liver function (ΔOFV −5.35; *p* = 0.021). All three covariates fulfilled the criteria of the backward analysis (*p* < 0.001). No significant influence of other covariates (e.g., age, sex, TBW, LBW, ABW, IBW, and BMI) on any of the PK parameters was found.

The typical MTT population values at pre-surgery, 7 days post-LSG, and 1 month post-LSG were estimated at 1.9, 0.5, and 0.9 h, respectively. The typical CL/F population value of CYP2C19 NMs with normal liver function was 16.7 L/h. The CL/F population values of CYP2C19 IMs and PMs were 0.8 and 0.34 times that of NMs, respectively. The CL/F population values of subjects with mild liver dysfunction was 0.6 times that of normal liver function. The Vd/F was estimated at 22.1 L. The population PK parameter estimates in the final model are shown in Table 2.

**Figure 1:**
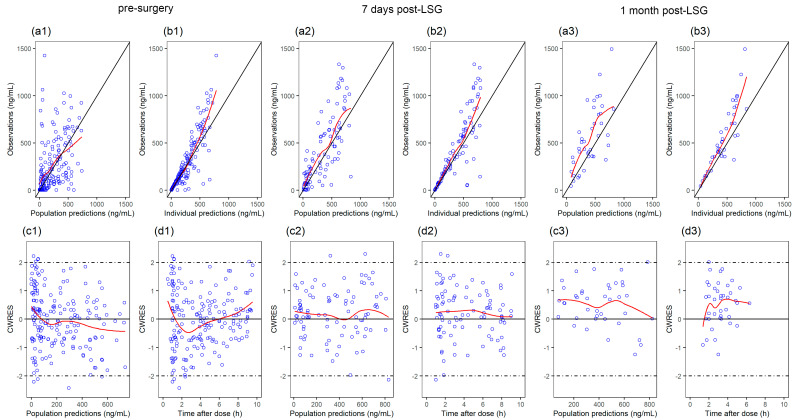
Goodness-of-fit plots for the final model at each visit: (**a1**,**a2**,**a3**) observations versus population predictions (PRED); (**b1**,**b2**,**b3**) observations versus individual predictions (IPRED); (**c1**,**c2**,**c3**) conditional weighted residuals (CWRES) versus PRED; (**d1**,**d2**,**d3**) CWRES versus time after dose.

**Figure 2:**
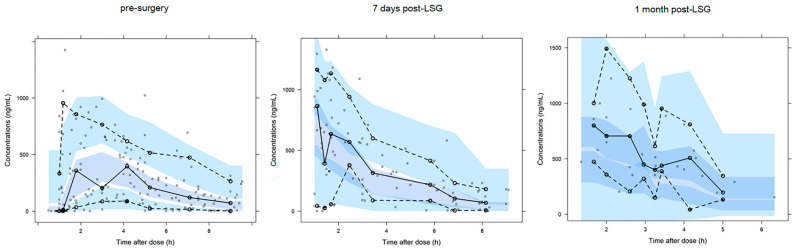
Prediction-corrected visual predictive check of the final model at each visit. Observed concentrations are indicated by black open circles, and the dotted and solid lines represent the 10th, 50th, and 90th percentiles of the observed data. The shaded areas represent 95% confidence intervals for the corresponding percentiles of simulated data.

Goodness-of-fit plots demonstrated that the final model appropriately described the observed data (Figure 1). Individual concentration–time curves of omeprazole at three visits were provided in the Supplementary Materials (Figure S1). The prediction-corrected VPC generally reflected a good agreement between the observations and simulations (Figure 2), indicating that the model adequately described the PK. In addition, all parameters estimates of final model fell near the median and within the 95% confidence interval of the SIR results (Table 2).

According to simulations, an important variability in T_max_ and C_max_ was observed before and after surgery (Table 3). LSG significantly affected the absorption rate of omeprazole, in which a reduced T_max_ and increased C_max_ were found 7 days and 1 month after surgery. However, omeprazole’s CL/F and AUC (area under the plasma concentration–time curve) were not different prior to surgery and post-LSG.

## 4. Discussion

Herein, we report the first study to investigate the PK profile of omeprazole in patients with obesity undergoing LSG. Using population PK modeling, we found the omeprazole absorption rate increased 7 days and 1 month after LSG, but the CL/F and exposure were unchanged. In addition, the influence of CYP2C19 genotype and liver function on omeprazole CL/F were observed, which was consistent with previously published studies [20,21].

**Figure 3:**
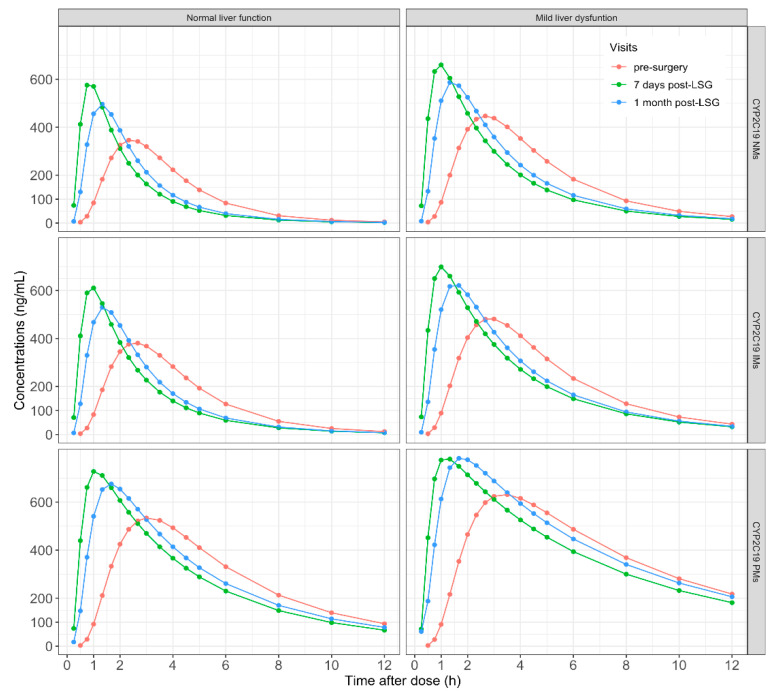
Mean concentration–time curves of omeprazole based on 1000 simulations stratified by liver function and CYP2C19 genotype in obese patients undergoing LSG.

Oral dosage forms of omeprazole are usually formulated as enteric-coated capsules or tablets. Under normal physiological conditions, after passing through the acidic environment in the stomach, the enteric coating dissolves and omeprazole is absorbed. After sleeve gastrectomy surgery, the enteric coating will dissolve faster due to the increase in gastric pH caused by the decrease of acid-producing gastric parietal cells. In addition, given that the gastric volume is greatly reduced, gastric emptying is accelerated, and the residence time of omeprazole in the stomach is shorter; hence it will reach the small intestine sooner, and the absorption can be completed in a shorter time after surgery. This could be observed from a significantly shorter T_max_ postoperatively (Table 3 and Figure 3). As there was no statistical difference in BMI between visit 1 and visit 2, the higher C_max_ was presumably caused by the faster absorption of omeprazole after LSG. Our results were also similar to other drug PK studies performed in obese patients undergoing LSG, showing a reduced T_max_ and increased C_max_ in operated patients compared with nonoperated individuals [9,28].

No effect of weight-derived indicators (e.g., TBW, LBW, and BMI) and visits on CL/F were found. Our previous work confirmed that omeprazole CL/F was decreased in obese adults versus normal-weight adults and speculated that CYP2C19 metabolic enzyme activity might be decreased in obese individuals caused by a low-grade inflammation state [20]. A recent research suggested that CYP2C19 activity is lower in patients with obesity versus nonobese controls and increased following RYGB-induced weight loss by measuring the plasma (3 h) 5-hydroxyomeprazole (5-OH-omeprazole)/omeprazole ratio [29]. These results indicated that the relationship between body weight and drug clearance is not always a simple allometric scaling in obese individuals. Interestingly, although we preformed PK sampling at a single dose before and 7 days after surgery and at repeat 1 month after surgery, we did not observe a difference in omeprazole CL/F between the three visits. Previous work suggested a significantly higher AUC and C_max_ after repeated omeprazole dosing in NMs but not in PMs [30,31]. This may provide some basis for explaining the unchanged clearance of omeprazole after single and multiple dosing in obese patients. As omeprazole is the inhibitor of the CYP2C19 metabolic enzyme, this leads to a decrease in first-pass effect in NMs due to auto-inhibition of CYP2C19 after repeated intake [31,32]. However, the CYP2C19 metabolic enzyme activity decreased in patients with obesity, and the auto-inhibition of CYP2C19 may be limited (similar to PMs); therefore, a comparable exposure was observed after single or repeated doses.

The CYP2C19 genotype was identified as a significant covariate for omeprazole CL/F, and a lower CL/F and higher AUC were observed in CYP2C19 IMs and PMs versus CYP2C19 NMs. Moreover, the CL/F was also found to decrease in patients with mild liver dysfunction. These results were reflected in PK parameters and concentration versus time curves obtained based on simulations (Table 3 and Figure 3). It has previously been established that the degree of acid inhibition by omeprazole was related to AUC [33,34]. However, there is no clear lower limit of efficacy at present. In the current analysis, the CL/F of omeprazole is not altered after short-term follow-up post-surgery. Furthermore, no heartburn, pantothenic acid, or other gastrointestinal symptoms were reported in patients followed up 1 month after surgery. Based on previous work, obese adults had a lower omeprazole CL/F and a higher AUC than normal adults [20]. Consequently, a 20 mg omeprazole dose daily may be adequate for obese patients undergoing LSG to relieve short-term gastrointestinal tract discomfort postoperatively.

There were some strengths and limitations in the current research. Firstly, our study had a relatively large study population, and the study design of repeated measures enabled obese subjects to act as their own control to minimize preoperative and postoperative variability. Secondly, omeprazole PK was not affected by interacting medication as we applied a specific exclusion criteria. A possible limitation was that the majority of obese patients included in the current analysis were female, but we did not observe a gender effect during model development, which was consistent with previous studies [35,36]. In addition, as the COVID-19 epidemic made it challenging to travel across provinces, quite a number of subjects could not participate in the PK follow up at visit 3. The changes in constituent ratio of CYP2C19 genotype at three visits may affect model stability; however, no statistical difference in constituent ratio of CYP2C19 genotype was observed among three visits using Fisher’s exact test (*p* = 0.471). Moreover, the estimated clearance ratios (CYP2C19 IMs: NMs or CYP2C19 PMs: NMs) were also similar to those reported in the literature [31]. Furthermore, the PK profile of omeprazole at 6–8 months after LSG was not studied in patients underwent LSG due to the difficulty of follow-up during the epidemic period. Therefore, the effect of significant weight loss in the middle postoperative period on the PK of omeprazole is still unclear. Previous studies have reported that significant weight loss postoperatively can reverse the decrease in CYP3A4 activity in obese patients and restore the enzyme activity to the same level as that of normal-weight individuals [37]. In addition, a recent study reported that the CYP2C19 activity increases with weight loss in obese patients after RYGB treatment. Therefore, the clearance of the CYP2C19 substrate in LSG patients may return to normal after notable weight loss, which deserves further investigation. Overall, the current study is innovative and of clinical significance, providing a specific reference for the postoperative use of omeprazole in LSG patients.

## 5. Conclusions

This study adequately characterized the effect of LSG on omeprazole PK in patients with obesity using nonlinear mixed-effect modeling. Our study showed faster absorption of omeprazole after LSG, but the CL/F and exposure were not different 7 days and 1 month after LSG compared with pre-surgery. We propose the use of 20 mg omeprazole once daily to relieve short-term discomfort symptoms of gastrointestinal tract postoperatively.

## Figures and Tables

**Table 1:** Demographic and clinical characteristics of study population.

Baseline Characteristics	Pre-Surgery (n = 62)	7 Days Post-LSG (n = 41)	1 Month Post-LSG (n = 20)
No. (%) of participants by sex			
Female	43 (69%)	29 (71%)	16 (80%)
Male	19 (31%)	12 (29%)	4 (20%)
No. (%) of participants by CYP2C19 genotype			
Normal metabolizers (NMs)	26 (42%)	17 (41.5%)	4 (20%)
Intermediate metabolizers (IMs)	27 (43.5%)	18 (44%)	12 (60%)
Poor metabolizers (PMs)	9 (14.5%)	6 (14.5%)	4 (20%)
Median (Q1~Q3) values for:			
Age (year)	31 (27~35)	28 (25~33)	28 (23~33)
Weight (kg)	110.8 (91~127)	114.6 (90.7~124.6)	* 96.2 (82.7~105.8)
Height (cm)	162.5 (158.6~167.5)	163 (158.5~169)	160.5 (158.1~166.4)
BMI (kg/m^2^)	40.3 (35~46)	41.2 (34.6~45.2)	* 36.1 (31.7~39.5)
LBW (kg)	55.1 (48.9~69.6)	55.3 (47~68.5)	* 50.9 (46~54)
IBW (kg)	54.8 (50.9~60.6)	54.8 (50.8~63)	52.7 (50.5~58.3)
ABW (kg)	78.5 (68.8~89.2)	79.2 (65.5~84.6)	* 71.7 (64.4~76)
No. (%) of participants by liver function			
Normal liver function	27 (43.5%)	6 (14.6%)	7 (35%)
Mild liver dysfunction	35 (56.5%)	35 (85.4%)	13 (65%)

BMI, body mass index; LBW, lean body weight; IBW, ideal body weight; ABW, adjusted body weight. * *p* < 0.001 from a Wilcoxon nonparametric paired test (visit 3 vs. visit 1) in obese subjects who completed both visit 1 and visit 3 (n = 20).

**Table 2:** Population pharmacokinetic parameter estimates for the final model and the SIR results.

Parameter	Final Estimates	RSE (%)	SIR (Median)	RSE (%)	95% CI
Structural model					
MTT for visit 1 (h)	1.91	6	1.92	6	1.68–2.13
MTT ratio for visit 2: visit 1 (*θ*_1_)	0.27	11.4	0.27	11.2	0.21–0.33
MTT ratio for visit 3: visit 1 (*θ*_2_)	0.45	7.6	0.45	7.6	0.38–0.51
CL/F for normal metabolizers with normal liver function (L/h)	16.7	14.9	16.7	14.4	11.9–21.3
CL/F ratio for CYP2C19 intermediate metabolizers: normal metabolizers (*θ*_3_)	0.8	12.4	0.8	12.3	0.6–0.98
CL/F ratio for CYP2C19 poor metabolizers: normal metabolizers (*θ*_4_)	0.34	17.8	0.35	17.5	0.22–0.45
CL/F ratio for mild liver dysfunction: normal liver function (*θ*_5_)	0.6	11.6	0.6	11.1	0.47–0.73
Vd/F (L)	22.1	5.5	22.2	5.4	19.7–24.5
Interindividual variability					
ω^2^ MTT	0.16	19.7	0.16	19.1	0.1–0.23
ω^2^ CL/F	0.16	22.8	0.15	21.6	0.1–0.22
IOV on CL/F	0.05	39.3	0.05	38.7	0.01–0.09
Residual error					
σ^2^ Proportional error	0.175	12.1	0.175	12.6	0.134–0.222

MTT, mean transit time; CL/F, apparent clearance; Vd/F, apparent volume of distribution; IOV, inter-occasion variability; SIR, sampling importance resampling; RSE, relative standard error; 95% CI, 95% confidence interval.

**Table 3:** Pharmacokinetic parameters for 20 mg omeprazole before and after LSG based on 1000 simulations.

	Pre-Surgery (N = 62)			7 Days Post-LSG (N = 41)			1 Month Post-LSG (N = 20)		
	CYP2C19 NMs	CYP2C19 IMs	CYP2C19 PMs	CYP2C19 NMs	CYP2C19 IMs	CYP2C19 PMs	CYP2C19 NMs	CYP2C19 IMs	CYP2C19 PMs
Normal liver function									
T_max_ (h)	2.3 (2~3)	2.3 (2~3.5)	2.7 (2~3.5)	* 0.8 (0.5~1)	* 0.8 (0.8~1)	* 0.8 (0.8~1)	* 1.3 (1~1.7)	* 1.3 (1~1.7)	* 1.3 (1~1.7)
C_max_ (ng/mL)	470 (386~540)	512 (427~588)	663 (595~717)	* 688 (634~738)	* 720 (660~763)	* 804 (771~830)	* 612 (547~674)	* 650 (583~709)	* 767 (720~809)
AUC_0-inf_ (ng·h/mL)	1198 (876~1609)	1476 (1097~2061)	3483 (2537~4697)	1185 (881~1585)	1540 (1067~2039)	3470 (2619~4562)	1156 (894~1613)	1497 (1128~2054)	3520 (2560~4725)
CL/F (L/h)	16.7 (12.4~22.8)	13.5 (9.7~18.2)	5.7 (4.3~7.9)	16.9 (12.6~22.7)	13 (9.8~18.7)	5.8 (4.4~7.6)	17.3 (12.4~22.4)	13.3 (9.7~17.7)	5.7 (4.2~7.8)
Mild liver dysfunction									
T_max_ (h)	2.7 (2~3.5)	2.7 (2~3.5)	3 (2.3~3.5)	* 0.8 (0.8~1)	* 0.8 (0.8~1)	* 1 (0.8~1)	* 1.3 (1~1.7)	* 1.3 (1~1.7)	* 1.3 (1~1.7)
C_max_ (ng/mL)	571 (501~640)	606 (533~670)	740 (682~782)	* 752 (708~789)	* 779 (734~808)	* 840 (817~858)	* 699 (637~744)	* 725 (674~768)	* 835 (788~894)
AUC_0-inf_ (ng·h/mL)	2032 (1484~2654)	2470 (1828~3242)	5948 (4376~8081)	1969 (1482~2654)	2530 (1882~3443)	5945 (4358~8258)	2027 (1519~2680)	2457 (1880~3345)	6019 (4399~8085)
CL/F (L/h)	9.8 (7.4~13.5)	8.1 (6.2~11)	3.4 (2.5~4.6)	10.2 (7.5~13.5)	7.9 (5.8~10.6)	3.4 (2.4~4.7)	9.9 (7.5~13.2)	8.1 (6~10.6)	3.3 (2.5~4.5)

Data are described as the median (Q1–Q3). NMs, normal metabolizers; IMs, intermediate metabolizers; PMs, poor metabolizers; T_max_, time to maximum plasma concentration; C_max_, maximum plasma concentration; AUC_0-inf_, area under the plasma concentration–time curve from time zero to infinity; CL/F, apparent clearance. * *p* < 0.001 vs. pre-surgery.

## Data Availability

The data presented in this study are available upon request from the corresponding author. The data are not publicly available due to the presence of ethical reasons as per local guidelines.

## Supplementary Materials

The following supporting information can be downloaded at: https://www.mdpi.com/article/10.3390/pharmaceutics14101986/s1, Figure S1: Individiual concentration-time curves of omeprazole in obese patients at three visits. Blue dots repesent observed concentrations, and grey and black lines repesent population predictions and individual predictions, respectively.
