# Supplementary material for: Effect of Laparoscopic Sleeve Gastrectomy on the Pharmacokinetics of Oral Omeprazole Using a Population Approach
Source: Pharmaceutics. 2022 Sep 20;14(10):1986. doi: 10.3390/pharmaceutics14101986 (PMC9608908; doi:10.3390/pharmaceutics14101986)
Supplement: Supplementary file 1 [file pharmaceutics-14-01986-s001.zip › Supplementary Material NONMEM code.pdf]

# NONMEM code

```
$SUBROUTINES ADVAN5
$MODEL

COMP=(1);DOSE
COMP=(2);CENTRAL
COMP=(3);transit com1
COMP=(4);transit com2
COMP=(5);transit com3
COMP=(6);transit com4
COMP=(7);transit com5
COMP=(8);transit com6
COMP=(9);transit com7
COMP=(10);transit com8
COMP=(11);transit com9
COMP=(12);transit com10
COMP=(13);transit com11
COMP=(14);transit com12


$PK
MTT= THETA(1)*EXP(ETA(1))                    ; Mean transit time
IF(VISIT.EQ.2) MTT=THETA(2)*MTT
IF(VISIT.EQ.3) MTT=THETA(3)*MTT

CL= THETA(4)*EXP(ETA(2)+IOVCL)                     ; Drug elimination clearance
IF(CYP.EQ.2) CL=THETA(5)*CL
IF(CYP.EQ.3) CL=THETA(6)*CL
IF(LF.EQ.2) CL=THETA(7)*CL

V=THETA(8)                                   ; Volume of the central compartment

VISIT1=0
IF(VISIT.EQ.1) VISIT1=1
VISIT2=0
IF(VISIT.EQ.2) VISIT2=1
VISIT3=0
IF(VISIT.EQ.3) VISIT3=1

IOVCL=VISIT1*ETA(3)+VISIT2*ETA(4)+VISIT3*ETA(5)    ; Inter-occasion variability of CL
```

```
NT=12                                   ; Number of transit compartments
KTR=(NT+1)/MTT                          ; Transit rate constant

K13=KTR
K34=KTR
K45=KTR
K56=KTR
K67=KTR
K78=KTR
K89=KTR
K910=KTR
K10T11=KTR
K11T12=KTR
K12T13=KTR
K13T14=KTR
K14T2=KTR

K20=CL/V
S2 = V/1000

$ERROR
DEL=0
IF (F.LE.0.0001) DEL=1
IPRE=F
W1= F
W2= 1
IRES= DV-IPRE
IWRE=IRES/(W1+W2)
Y = F + W1*ERR(1)
```
