# Supplementary material for: Effect of Laparoscopic Sleeve Gastrectomy on the Pharmacokinetics of Oral Omeprazole Using a Population Approach
Source: Pharmaceutics. 2022 Sep 20;14(10):1986. doi: 10.3390/pharmaceutics14101986 (PMC9608908; doi:10.3390/pharmaceutics14101986)
Supplement: Supplementary file 1 [file pharmaceutics-14-01986-s001.zip › Supplementary Materials Figure S1.pdf]

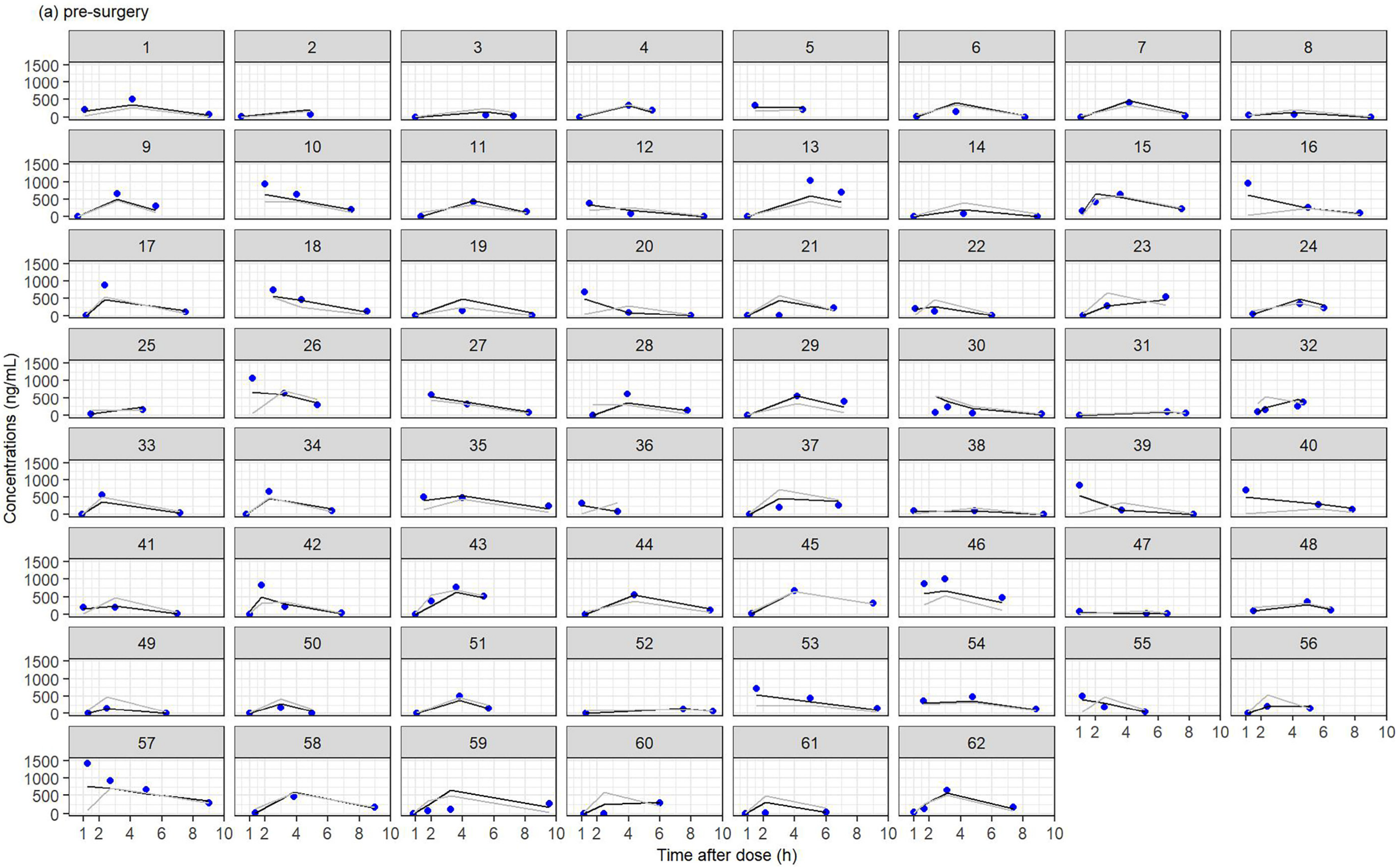
(a) pre-surgery
Concentrations (ng/mL)
Time after dose (h)

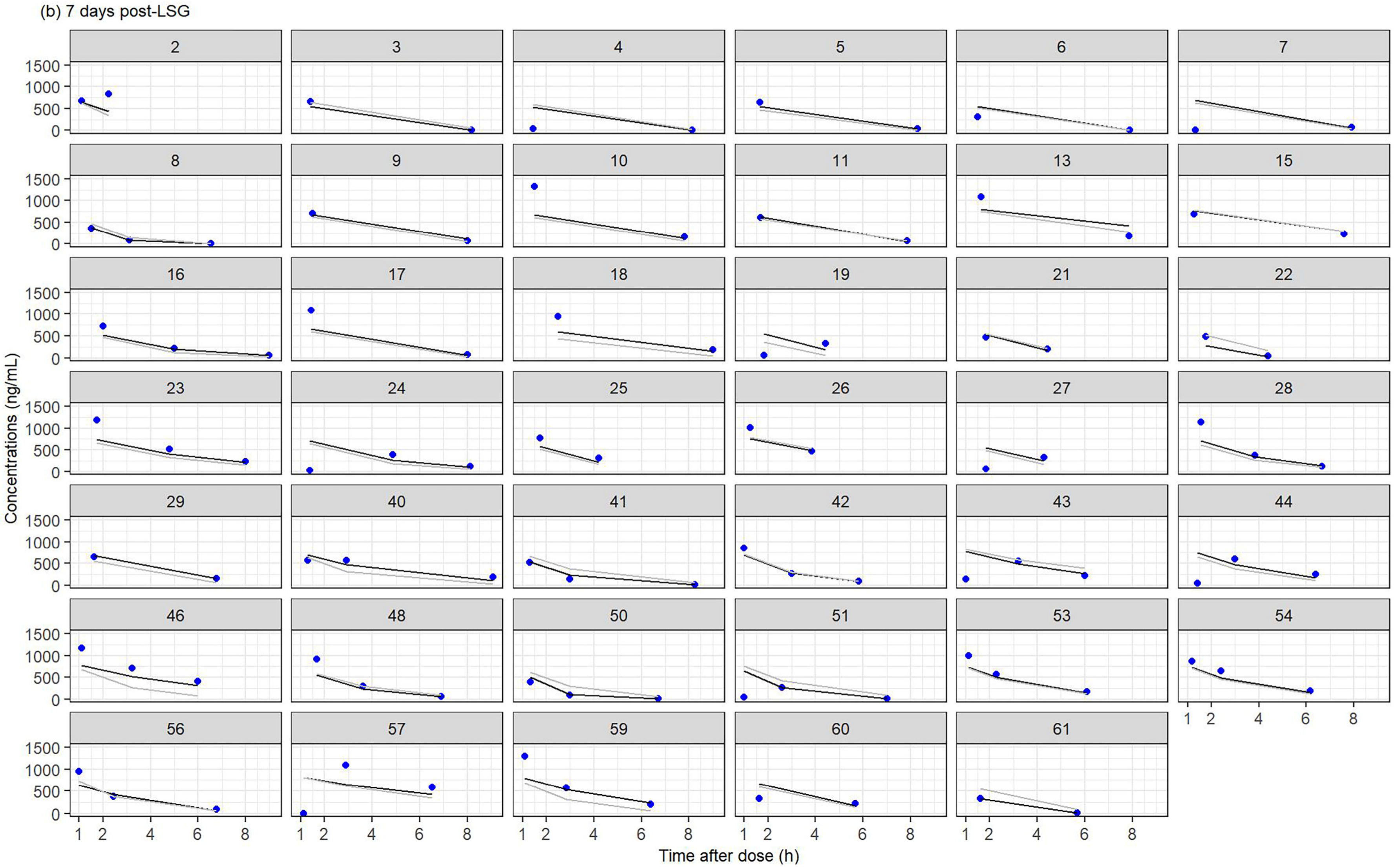
(b) 7 days post-LSG
2
3
4
5
6
7
8
9
10
11
13
15
16
17
18
19
21
22
23
24
25
26
27
28
29
40
41
42
43
44
46
48
50
51
53
54
56
57
59
60
61
1500
1000
500
0
1
2
4
6
8
Concentrations (ng/mL)
Time after dose (h)

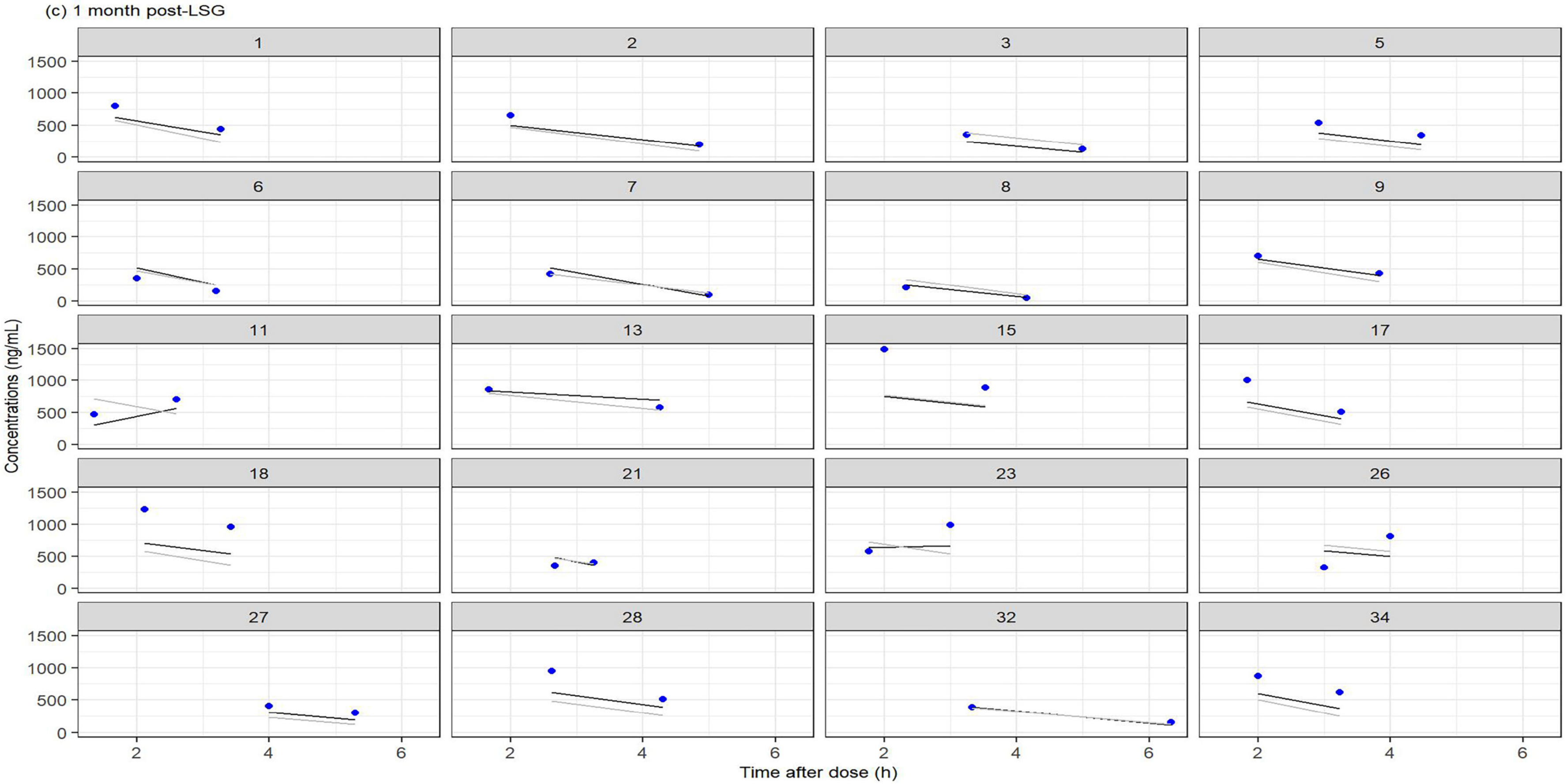

Figure S1. Individiual concentration-time curves of omeprazole in obese patients at three visits. Blue dots repesent observed concentrations, and grey and black lines repesent population predictions and individual predictions, respectively.
